# Primary aortoenteric fistula: a case report and literature review

**DOI:** 10.1097/RC9.0000000000000083

**Published:** 2026-01-28

**Authors:** Pietro Fransvea, Tommaso Partipilo, Tommaso Donati, Gabriele Sganga, Sergio Alfieri, Fausto Rosa

**Affiliations:** a UOC Chirurgia d’Urgenza e del Trauma, Fondazione Policlinico Universitario A. Gemelli, IRCCS - Università Cattolica del Sacro Cuore, Rome, Italy; b UOC Chirurgia Vascolare, Fondazione Policlinico Universitario A. Gemelli, IRCCS, Rome, Italy

**Keywords:** aortoduodenal fistula, emergency surgery, gastrointestinal bleeding, no aneurysm, ovarian cancer, primary aortoenteric fistula

## Abstract

**Introduction:**

Primary aortoenteric fistulas are rare, life-threatening conditions, usually secondary to abdominal aortic aneurysms. Primary aortoduodenal fistula (PADF) without aneurysmal disease is extremely rare, with very few cases reported in the literature. Delayed diagnosis is common and contributes to high mortality.

**Case presentation:**

We report the case of a 71-year-old woman with high-grade serous ovarian cancer who developed a PADF in the absence of an aortic aneurysm. The patient presented with hematemesis and abdominal pain. Initial investigations, including Computed Tomography (CT) scan and upper endoscopy (EGDS), were inconclusive. Although she was initially stabilized, she experienced recurrent gastrointestinal bleeding leading to hemodynamic deterioration. Emergency CT showed active arterial hemorrhage near the duodenum. An urgent exploratory laparotomy confirmed the presence of a PADF, which was surgically repaired using a bovine pericardial patch. Despite surgical intervention, the patient succumbed to postoperative multiorgan failure.

**Clinical discussion:**

PADFs most frequently involve the third or fourth portion of the duodenum and are strongly correlated with aneurysmal disease. However, rare etiologies such as radiation therapy, malignancy, or infection may precipitate fistula formation even in the absence of aneurysms. Our literature review identified only 16 similar cases reported since 2015. Diagnosis remains challenging due to non-specific symptoms and inconclusive early imaging. A high index of suspicion is essential. CT angiography is the most effective diagnostic modality. Definitive management requires urgent surgical or endovascular repair, although prognosis remains poor in hemodynamically unstable patients.

**Conclusion:**

This case underscores the need to consider PADF in the differential diagnosis of gastrointestinal bleeding, even in the absence of aneurysmal disease, particularly among oncologic patients with prior radiation exposure or retroperitoneal inflammation. Early diagnosis, prompt imaging, and a multidisciplinary approach are essential to improving patient outcomes.

## Introduction

An aortoenteric fistula is a rare and life-threatening condition, with mortality rates nearing 100% if not promptly diagnosed and treated. Primary aortoenteric fistula (PAEF) refers to the spontaneous formation of a pathological communication between the aorta and the gastrointestinal tract, unassociated with prior vascular surgery. It is most commonly linked to abdominal aortic aneurysms (AAA), in which chronic pressure and inflammation cause erosion into adjacent bowel loops. The incidence of PAEF is estimated at 0.04%–0.07% in the general population and complicates up to 2% of AAA cases^[^1^]^. However, primary aortoduodenal fistula (PADF) in the absence of an aortic aneurysm represents an extremely rare entity.

Since 2015, our review of the literature has identified only 16 such cases (Table 1). These rare occurrences are often secondary to other predisposing factors such as radiation therapy, malignancy, infection, foreign bodies, or prior retroperitoneal surgery^[^2,3^]^. The diagnostic process remains challenging due to the rarity of the condition and the non-specific nature of clinical presentations, with the classic triad of gastrointestinal bleeding, abdominal pain, and a pulsatile abdominal mass being present in fewer than 11% of cases^[^4^]^.

**Table 1:** Literature review since 2015.

Author	Year	Country	Age	Sex	Symptoms	Diagnosis	Location	Cause	Laparotomy	Endovascular treatment	Outcome
Gordon *et al*^[^5^]^	2016	UK	59	F	Hematemesis, abdominal pain	Endoscopy	D4	Retroperitoneal mass (poorly differentiated carcinoma)	Yes	No	Death postoperatively
Naranje *et al*^[^6^]^	2016	India	46	F	Melena, abdominal pain, vomiting	CT	D4	Not identified	No	No	Death pre-diagnosis
Liao *et al*^[^7^]^	2017	Canada	47	M	Septic shock	CT	D3	Foreign body (toothpick in the posterior wall of the duodenum)	Yes	No	Alive at 3y FU
Morikawa *et al*^[^4^]^	2017	Japan	56	M	Hematemesis, abdominal pain	Endoscopy, CT, angiography	Not identified	Radiation therapy	No	Yes	Death 33d after procedure (aspiration pneumonia)
Dimech et al*^[^8^]^	2018	Malta	75	M	Hematemesis	Endoscopy, CT, angiography	D2-D3	Radiation therapy? Retroperitoneal lymph node metastasis (right colonic cancer)?	No	Yes	Death 3 m after procedure (re-bleeding)
Brough *et al*^[^9^]^	2019	Australia	70	M	Hematemesis	Endoscopy, CT	D4	Not identified	Yes	Yes	Not reported
Fuchigami *et al*^[^10^]^	2020	Japan	76	M	Hematemesis, melena	Endoscopy, CT, angiography	D3	Not identified	No	Yes	Alive at 11 m FU
Osella *et al*^[^11^]^	2020	Italy	69	M	Hematemesis, melena	Endoscopy, CT, angiography	Not reported	Atherosclerotic ulcer	Yes	Yes	Death after procedure (time not reported)
Özdemir et al* ^[^12^]^	2020	Turkey	56	F	Melena	CT scan	D3	Previous surgery (retroperitoneal lymph node dissection 4y earlier)	Yes	No	Alive at 15 m FU
Sakurai *et al*^[^13^]^	2021	Japan	48	M	Hematochezia, hypovolemic shock	CT scan, angiography	D3	Retroperitoneal lymph node metastasis (testicular cancer)	No	Yes	Death 18d after procedure (massive bleeding)
Stuart *et al*^[^14^]^	2021	USA	58	M	Hematemesis	CT scan, endoscopy	D4	Duodenal stent	No	No	Death after diagnosis
Bacopanos *et al*^[^15^]^	2022	Australia	51	M	Hematemesis, shock	CT scan, angiography	Not reported	Duodenal stent	No	Yes	Death 2y after procedure (cause unknown)
			59	F	Intestinal bleeding	CT scan, endoscopy, angiography	Not reported	Duodenal stent	No	Yes	Death 3d after procedure (sepsis)
			73	F	Hematemesis	CT scan, angiography	Not reported	Duodenal stent	No	Yes	Alive at 3 m FU
Kehagias *et al*^[^16^]^	2022	Greece	58	F	Hematemesis, hematochezia, hemodynamic instability	Endoscopy, CT scan, angiography	D3	Duodenal stent	No	Yes	Alive at 10 m FU
Vacca *et al*^[^17^]^	2022	Italy	74	M	Rectal bleeding	Endoscopy, CT scan, angiography	D4	Atherosclerosis?	Yes	No	Death postoperatively
Corrêa *et al*^[^18^]^	2023	Brazil	39	Not repoerted	Hematemesis, back pain	Endoscopy, CT scan, angiography	D3	Retroperitoneal lymph node metastasis (Hodgkin Lymphoma)	No	Yes	Alive at 4 m FU
Aroudam *et al*^[^19^]^	2025	Morocco	72	M	Hematemesis, melena, abdominal pain	CT scan	D3-D4 junction	Not identified	No	No	Death before any intervention/therapeutic management
Sassi *et al*^[^20^]^	2025	Morocco	39	F	Abdominal pain, melena	Endoscopy, CT scan	D3	Inflammatory aortitis	Yes	No	Not reported
Our case	2024	Italy	71	F	Hematemesis, abdominal pain	Endoscopy, CT	D4	Radiation therapy?	Yes	No	Death postoperatively

* Cases of fistula between duodenum and proximal common iliac artery (right in Dimech *et al* and left in Özdemir *et al*)

Here, we present a rare case of PADF in a 71-year-old woman with high-grade serous ovarian cancer (HGSOC), occurring in the absence of aortic aneurysm or prior vascular reconstruction, and discuss the clinical implications of such atypical presentations.

## Case presentation

A 71-year-old Caucasian woman with a history of hypertension and newly diagnosed HGSOC in July 2021 underwent neoadjuvant chemotherapy followed by cytoreductive surgery and adjuvant chemotherapy. Complications from her treatments included the development of an entero-vaginal fistula requiring surgical intervention with ileostomy in October 2021. In May 2023, she received SBRT for lymphadenopathies, followed by second-line chemotherapy and bevacizumab maintenance therapy from January 2024 onward.

In April 2024 during a routine follow-up, a CT scan incidentally identified a retroperitoneal perforation near the distal duodenum with an adjacent small abscess near the superior mesenteric artery (SMA). The patient remained asymptomatic and was managed conservatively, being discharged after a brief hospital stay.

In May 2024, she presented to the Emergency Department with coffee ground vomitus and signs of septic shock. Initial investigations, including CT scan and upper GI series, showed no significant abnormalities, and she was discharged after stabilization.

However, in July 2024, she returned with hematemesis and mild abdominal pain. Laboratory tests revealed anemia (hemoglobin 8.9 g/dL), leukocytosis (14.58 × 10^9/L), and thrombocytosis (540 × 10^9/L), along with elevated lactate levels (5.7 mmol/L). A repeat CT scan indicated an increase in the size of the abdominal collection near the duodenum and SMA, without active bleeding identified. Within 24 hours, she deteriorated rapidly with worsening leukocytosis (30.13 × 10^9/L) and decreased hemoglobin (6.7 g/dL). EGDS identified blood clots and mucosal discontinuity in the distal duodenum, managed with adrenaline injection and clipping, but bleeding recurred. The patient was transferred to the ICU but deteriorated further, demonstrating hemodynamic instability. A subsequent CT scan (Figure) revealed copious arterial bleeding from an aortoduodenal fistula, prompting emergent laparotomy. During surgery, challenging adhesiolysis and a transverse colon section were required. The vascular surgeon repaired the fistula using a bovine pericardial patch, chosen for its superior biocompatibility, flexibility, and resistance to infection compared to synthetic grafts in contaminated fields. Despite exhaustive efforts, including intensive resuscitation and surgical repair, the patient succumbed to multiorgan failure in the postoperative period.

**Figure 1.**
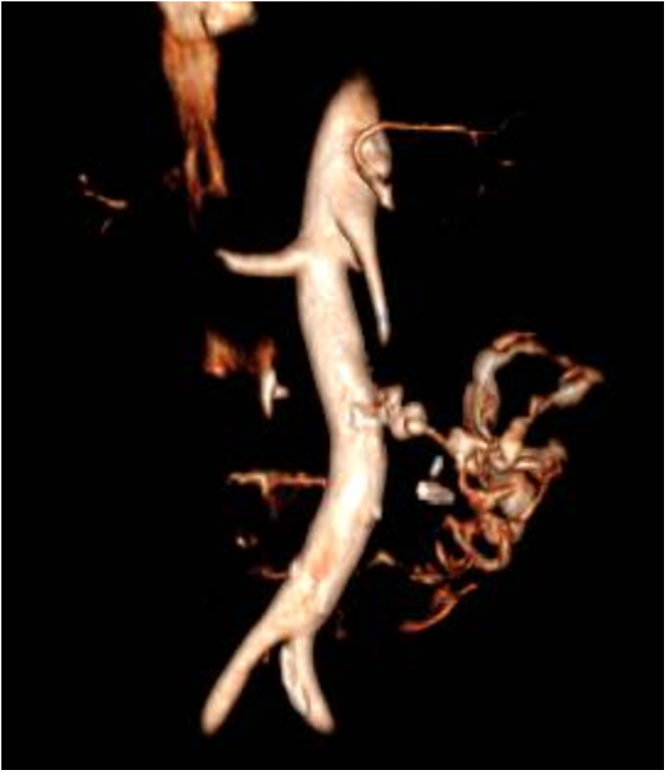
CT scan showing a retroperitoneal perforation near the distal duodenum with an adjacent small abscess near the superior mesenteric artery (SMA).

Table 2 summarizes the sequence of investigations and interventions leading to the diagnosis and surgical management of the primary aortoduodenal fistula.

**Table 2:** Diagnostic and therapeutic timeline summarizing the sequence of investigations and interventions leading to the diagnosis and surgical management of the primary aortoduodenal fistula.

Date/period	Clinical event	Investigations and findings	Interventions/outcome
April 2024	Routine oncologic follow-up	CT scan revealed retroperitoneal perforation near distal duodenum with small adjacent abscess; no bleeding detected.	Conservative management and discharge.
May 2024	First acute episode: coffee-ground vomitus, septic shock	CT and upper GI series inconclusive; no active bleeding.	Supportive therapy and discharge after stabilization.
July 2024 (Day 1)	Recurrent hematemesis and abdominal pain	Hb 8.9 g/dL, WBC 14.6 × 10^9^/L, lactate 5.7 mmol/L. CT showed enlarged collection near duodenum and SMA but no contrast leak.	Initial stabilization.
July 2024 (Day 2)	Rapid deterioration	Hb 6.7 g/dL, WBC 30.1 × 10^9^/L. EGDS showed blood clots and mucosal discontinuity in duodenum, treated with adrenaline and clipping.	Transient control of bleeding.
July 2024 (Day 3)	Hemodynamic collapse	CT angiography showed active arterial extravasation near third duodenal portion – consistent with PADF.	Emergency laparotomy.
Intraoperative	Extensive adhesions, retroperitoneal inflammation	Aortoduodenal fistula confirmed.	Aortic repair using bovine pericardial patch performed by vascular surgeon.
Postoperative period	Persistent shock and organ dysfunction	—	Death due to multiorgan failure despite maximal resuscitation efforts.

## Discussion

PADF is a rare and life-threatening vascular-gastrointestinal emergency. Traditionally, PADF has been strongly associated with abdominal aortic aneurysms (AAA), which account for the vast majority of PAEFs. In these cases, chronic mechanical stress, wall inflammation, and proximity between the aneurysm and the duodenum lead to erosion and fistulization^[^1^]^. However, the occurrence of PADF in the absence of aneurysmal disease is exceptionally rare. Our review of the literature identified only 16 published cases of non-aneurysmal PADF between 2015 and 2025. These cases – summarized in Table 1 – highlight the broad range of predisposing factors other than AAA, including malignancy, radiotherapy, retroperitoneal infection or inflammation, foreign body ingestion, prior surgery, or duodenal stenting. In some reports, no identifiable cause was found^[^2-4^]^. In our case, the patient had undergone multiple oncologic treatments, including SBRT (stereotactic body radiotherapy) for para-aortic lymphadenopathies, systemic chemotherapy, and bevacizumab maintenance therapy. Bevacizumab, a VEGF (vascular endothelial growth factor) inhibitor, is associated with impaired vascular healing and increased risk of gastrointestinal perforation or fistula formation^[^21^]^. Radiation therapy is known to induce delayed tissue necrosis, fibrosis, and microvascular damage, which may contribute to aortic wall fragility and predispose to fistula development, even years after exposure^[^22^]^. Several authors have emphasized the diagnostic challenges of PADF. The classic triad of gastrointestinal bleeding, abdominal pain, and a pulsatile abdominal mass is observed in fewer than 11% of patients^[^23^]^. In practice, intermittent “herald” bleeding is often misinterpreted, delaying the diagnosis. In a systematic review by Saers and Scheltinga^[^2^]^, it was noted that diagnostic delay significantly increases mortality and that high clinical suspicion is crucial, especially when imaging or endoscopy is inconclusive. In our patient, initial CT imaging showed a retroperitoneal perforation near the duodenum and SMA, but no active bleeding. This was followed by multiple episodes of hematemesis and transient improvement. Endoscopy, though performed twice, failed to identify the fistula, likely due to the intermittent nature of bleeding and the difficulty in visualizing the distal duodenum. Liao *et al*^[^7^]^ described a similar diagnostic dilemma in a young male patient, where a toothpick caused delayed duodenal perforation into the aorta, and only repeat CT and high suspicion led to diagnosis^[^24^]^. CT angiography is currently considered the gold standard for diagnosis, with sensitivity as high as 94% in some studies^[^5^]^. It allows visualization of contrast extravasation, perigraft air (in post-operative cases), or aortoduodenal wall defects. However, as seen in our case and in others, even CT may initially fail to detect the fistula, especially if bleeding is intermittent or temporarily tamponade^[^5,6^]^. The therapeutic approach is highly dependent on patient stability and institutional resources. Surgical repair remains the cornerstone of treatment, particularly in patients with generalized peritonitis or retroperitoneal sepsis. Our case required urgent laparotomy with aortic repair using a bovine pericardium patch – an approach described by other authors in high-risk or infected fields^[^25^]^. However, outcomes remain poor in emergency open procedures, with reported mortality rates exceeding 80%. Endovascular aneurysm repair (EVAR) has emerged as an alternative in selected cases, offering shorter operative times and less physiological stress. In the review by Leon and Mills^[^3^]^, EVAR showed improved short-term survival, but the risk of infection and rebleeding remains significant if the duodenal defect is not adequately managed. Notably, in aneurysm-free PADF cases, the anatomy may not permit stent grafting, or infection may contraindicate its use, as occurred in several cases from our review. The literature continues to debate the best approach. Morikawa *et al*^[^4^]^ favored endovascular-first strategies for stabilization, followed by delayed definitive surgery in selected cases. Conversely, Kehagias *et al*^[^16^]^ emphasized the importance of radical debridement and open repair to reduce late complications, particularly in infected case. Ultimately, this case contributes to a growing body of literature on non-aneurysmal PADF – a subset of aortoenteric fistulas that remains underrecognized and underreported. The absence of an aneurysm may paradoxically delay diagnosis, as clinicians often exclude PADF from the differential when no aortic dilation is observed. However, as this case and others demonstrate, local oncologic or inflammatory processes alone can suffice to create a fistulous tract between the aorta and duodenum^[^8-15,17–20^]^. This case fits within the growing subset of non-aneurysmal PADFs where malignancy, radiotherapy, and vascular-targeted therapy act as synergistic triggers. Compared with previously reported cases, our patient’s presentation was consistent with the gradual evolution of radiation- and drug-induced vascular fragility. However, unlike most published cases, the diagnosis was delayed despite multiple CT and endoscopic evaluations, emphasizing the subtlety of early signs. This reinforces the necessity of maintaining PADF in the differential even when imaging is initially non-diagnostic.

**HIGHLIGHTS**

- We report a rare case of primary aortoduodenal fistula (PADF) occurring without an aortic aneurysm.
- Only 16 similar cases without aneurysmal disease have been reported since 2015.
- The PADF was associated with radiation therapy and malignancy in an oncologic patient.
- Diagnosis was delayed due to intermittent symptoms and inconclusive imaging.
- High clinical suspicion and timely surgical intervention are crucial despite poor prognosis.

## Methods

This work has been reported in line with the SCARE, attached as supplementary material^[^26^]^. This case report was conducted in accordance with the Declaration of Helsinki and institutional ethical standards. Formal approval was waived given the single-patient, retrospective, and non-interventional nature of the report. The authors confirm that patient confidentiality was maintained throughout, and no identifying information has been disclosed.

## Conclusion

This case underscores the diagnostic and therapeutic challenges of PADF, particularly in the absence of an abdominal aortic aneurysm – a presentation reported in only a limited number of cases. The patient’s oncological history and prior treatments likely contributed to local inflammation and subsequent fistula formation. Clinicians should maintain a high index of suspicion for PADF in patients presenting with unexplained upper gastrointestinal bleeding, even in the absence of typical risk factors. Early recognition through appropriate imaging and prompt multidisciplinary intervention are crucial to improving survival in this otherwise frequently fatal condition. This case therefore reinforces the need for heightened clinical vigilance and rapid, coordinated management to optimize patient outcomes

## Research registration unique identifying number (UIN)

1. Name of the registry: Università Cattolica - Fondazione Policlinico Universitario A. Gemelli IRCCS.

2. Unique identifying number or registration ID: 2024/648073.

3. Hyperlink to your specific registration (must be publicly accessible and will be checked):
